# Ethnic differences in the effect of environmental stressors on blood pressure and hypertension in the Netherlands

**DOI:** 10.1186/1471-2458-7-118

**Published:** 2007-06-23

**Authors:** Charles Agyemang, Carolien van Hooijdonk, Wanda Wendel-Vos, Joanne K Ujcic-Voortman, Ellen Lindeman, Karien Stronks, Mariel Droomers

**Affiliations:** 1 Centre for Prevention and Health Services Research; National Institute for Public Health and the Environment, PO Box 1, 3720 BA Bilthoven, The Netherlands; 2 Dept of Social Medicine, Academic Medical Centre, University of Amsterdam, The Netherlands; 3 Dept of Epidemiology, Documentation and Health Promotion, GGD Amsterdam, Amsterdam, The Netherlands; 4 Department of Research and Statistics, City of Amsterdam, the Netherlands

## Abstract

**Background:**

Evidence strongly suggests that the neighbourhood in which people live influences their health. Despite this, investigations of ethnic differences in cardiovascular risk factors have focused mainly on individual-level characteristics. The main purpose of this study was to investigate associations between neighbourhood-level environmental stressors (crime, housing density, nuisance from alcohol and drug misuse, quality of green space and social participation), and blood pressure (BP) and hypertension among different ethnic groups.

**Methods:**

Individual data from the Amsterdam Health Survey 2004 were linked to data on neighbourhood stressors creating a multilevel design for data analysis. The study sample consisted of 517 Dutch, 404 Turkish and 365 Moroccans living in 15 neighbourhoods in Amsterdam, the Netherlands.

**Results:**

Amongst Moroccans, high density housing and nuisance from drug misuse were associated with a higher systolic BP, while high quality of green space and social participation were associated with a lower systolic BP. High level of nuisance from drug misuse was associated with a higher diastolic BP. High quality of green space was associated with lower odds of hypertension. Amongst Turkish, high level of crime and nuisance from motor traffic were associated with a higher diastolic BP. Similar associations were observed among the Dutch group but none of the differences were statistically significant.

**Conclusion:**

The study findings show that neighbourhood-level stressors are associated with BP in ethnic minority groups but were less evident in the Dutch group. These findings might imply that the higher BP levels found in some ethnic minority groups might be partly due to their greater susceptibility to the adverse neighbourhood environment in which many ethnic minority people live. Primary prevention measures targeting these neighbourhood stressors may have an impact in reducing high BP related morbidity and mortality among ethnic minority groups.

## Background

Cardiovascular disease (CVD) is the leading cause of death in industrialised countries. High blood pressure (BP) is one of the important causes of cardiovascular diseases and its role is set to continue [1]. The risk of cardiovascular disease associated with high BP is consistent and independent of other risk factors [2]. The high prevalence of hypertension is well reflected in the high prevalence of stroke and cardiovascular disease across the globe [3]. In western societies, BP levels and prevalence of hypertension differ by ethnic group with most studies showing higher levels and rates in the ethnic minority groups than in the European populations [4-7]. The explanations for the higher BP levels and the higher prevalence of hypertension in ethnic minority populations still remain unclear [8].

As in most CVD epidemiology, investigations of high BP in ethnic groups have focused mainly on individual level characteristics such as obesity, education and genes [9,10]. The environmental effect on BP and hypertension in different ethnic groups has hardly ever been examined. Evidence strongly suggests that the neighbourhood in which people live influences their health, either in addition to or in interaction with individual level characteristics [11]. A systematic review of multilevel studies [12], for example, showed fairly consistent and modest neighbourhood effects on health despite the differences in study designs, neighbourhood measures and possible measurement errors. More recently, adverse neighbourhood factors have also shown to be positively associated with coronary heart disease (CHD) [15,16] and insulin resistance syndrome [17].

There are also indications that the impact of the neighbourhood environment on ill health is greater in ethnic minority population groups than in European populations [13,14]. For example, Cubbin and colleagues' study showed a stronger neighborhood deprivation effect on cardiovascular risk factors in African Americans than in White Americans [14].

There are several mechanisms through which neighbourhood environment may be linked to the development of high BP, for example, through their influence on health related behaviours or through psychosocial pathways. Recent studies indicate a possible role of neighbourhood environments in influencing physical activity [18-21] and diet [19,22], both of which may be related to high BP [23]. It has been shown that neighbourhoods characterised by poor physical quality are associated with psychosocial stress [24]. Social participation may also have direct effects on health outcomes by influencing a series of physiologic pathways or via social influence or supportive functions that influence health-promoting or health-damaging behaviors [25]. Living in a stressful neighbourhood may discourage residents from taking up important lifestyle measures such as physical activity which, in turn, may lead to the development of high BP. It is also possible that the biological pathway between these neighbourhoods' factors and BP may be mediated by an abnormal neuroendocrine secretory pattern [26] due to stress. Neighbourhood stressors may vary between neighbourhoods, which may lead to differences in development of high BP.

Perception of environmental stressors may differ between different ethnic groups due to differences in culture, language, migration history and socio-economic positions [23,27]. Neighbourhood stressors may therefore provide important clues for explaining the higher BP levels and hypertension rates in ethnic minority populations since many of these populations live in disadvantaged neighbourhoods with high levels of stress. The main objective of this paper was to determine whether neighbourhood environmental stressors were associated with BP and hypertension in Dutch, Turkish and Moroccan ethnic groups in Amsterdam, the Netherlands.

Turkish and Moroccans are two of the largest ethnic minority groups in the Netherlands. They came to the Netherlands in the 1960s and early 1970s as labour migrants. The initial period of labour migration was followed by a period in which many guest workers brought their spouses and children over to the Netherlands. A large percentage of Turkish and Moroccan especially first generation immigrants have lower educational levels, poor Dutch language proficiency, and tend to stay within their own culture [28].

## Methods

The data in this study were collected at two levels. The individual (first) level included information on demographics, body mass index (BMI), BP and hypertension. The contextual (second) level included information on environmental stressors. These two levels were linked by neighbourhood, creating a multilevel design for data analysis.

### Data collection at the individual level

**Figure 1:**
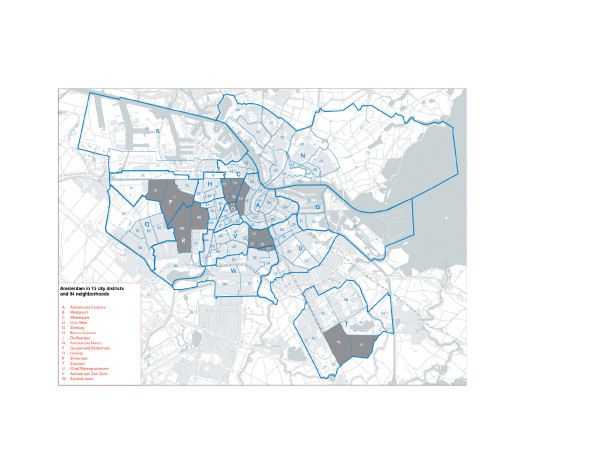


The individual level data came from the Amsterdam Health Survey 2004. This cross-sectional study was carried out by Amsterdam Municipal Health Service (GGD Amsterdam) in collaboration with the National Institute for Public Health and Environment (RIVM) to monitor the health of the Amsterdam general population aged ≥18 years. The study sample was drawn from the Amsterdam municipal registers in five city districts in Amsterdam (Figure 1). The population of these districts combined is representative for the total population of Amsterdam. The sample was stratified by ethnicity and five age groups (18–34 years, 35–44 years, 45–54 years, 55–64 years and 65 years or older). Within each stratum a random sample was drawn. The Turkish and Moroccan ethnic groups were oversampled to ensure sufficient numbers of people from these groups. This method was necessary to boost Turkish and Moroccan ethnic groups in the sample because of their relatively low representation in the total population and their lower participation rate in national and local surveys in the Netherlands. In 2004, the people in the sample were invited for an interview and medical examination in a community health centre. All interviews were conducted in the language of choice of the respondent (i.e., Dutch, Turkish, Moroccan-Arabic or Berber). The final response rate was 44% (Dutch 46%, Turks 50% and Moroccans 39%). Data were weighted to correct for oversampling by ethnic groups. All participants signed a consent form. The Medical Ethical Committee of the Amsterdam Medical Centre approved the study protocols.

### Individual level variables

Ethnicity was classified according to the self-reported country of birth and/or the country of birth of the respondent's mother or father. Ethnicity refers to the group individuals belong to as a result of their culture, which includes language, religion, diet and ancestry [29]. The term 'Moroccan' refers to people, and their offspring who migrated to the Netherlands via Morocco. The term 'Turkish' refers to people, and their offspring who migrated to the Netherlands via Turkey. The term 'Dutch' refers to people with Dutch European ancestral origin.

Blood pressure was measured with a validated oscillometric automated digital device (OMRON HEM-711). Using appropriate cuff sizes, two readings were taken on the left arm in a seated position after the subject had been seated for at least five minutes. Trained nurses performed all the medical examinations. The mean of the two readings was used for analysis. Hypertension was defined as SBP ≥ 140 mm Hg, or DBP ≥ 90 mm Hg, or being on anti-hypertensive therapy. Education level was determined during the interview. Body mass index (BMI) was calculated as weight (kg) divided by height (m^2^).

### Data collection at the contextual level

All the contextual level variables originated from three different data sources (Living in Amsterdam Survey 2003, Amsterdam Living and Security Survey 2004, and The Social State of Amsterdam City Survey 2004) and were provided by the Department of Research and Statistics of Amsterdam Municipality (O+S Amsterdam). The aggregated data were dichotomised (coded low = 0 and high = 1), with low representing the eight neighbourhoods with the lowest scores and high representing the other seven neighbourhoods with the highest scores (Table 1).

**Table 1:** Distribution of neighbourhood attributes for the 15 neighbourhoods

	**Low**	**High**	**P-value**
% Crime			
Mean Min-max	51.9 38.5–55.6	57.2 56.0–60.6	.001
			
% Housing density			
Mean Min-max	45.5 32.3–57.7	76.1 61.4–87.4	.001
			
% Nuisance from motor traffic			
Mean Min-max	25.2 6.7–36.7	52.2 38.8–87.0	.001
			
Mean Quality of green facilities			
Mean Min-max	5.9 5.5–6.28	6.9 6.4–7.2	.001
			
% Nuisance from alcohol misuse			
Mean Min-max	5.1 0.9–13.7	27.7 19.50–40.5	.001
			
% Nuisance from drug misuse			
Mean Min-max	11.8 6.2–16.8	30.5 19.2–41.1	.001
			
% Social participation			
Mean Min-max	37.5 25.2–41.8	44.6 44.6–69–20	.001

### Neighbourhood environmental stressor variables

We calculated the proportion of people in each neighbourhood who reported experience of crime (such as break-ins, theft, aggravated assault, vandalism and stolen purse) in the past 12 months, being bothered by excessive motor traffic, nuisance from frequent alcohol and drug misuse, living in a neighbourhood with cramped housing (housing density), involvement in at least one of the activities of formal or informal organisations (sports clubs, nature or animal organisations, political, women or ethnic minority organisations, union meeting, theatre/cinema, arts exhibition, church, youth organisations, library and meeting of other organisations) and quality of green space. Quality of green space was based on a scale of 1 to 10 (from very ugly to very beautiful). We calculated the mean score for each neighbourhood. To allow for the non-linear effects, and the relative smaller number of neighbourhoods, the neighbourhood-level stressor variables were dichotomised for each ethnic group. In the Netherlands, neighbourhoods are areas with a similar type of building, often delineated by natural boundaries. As a result, they are socio-culturally quite a homogenous group [30].

### Data Analysis

Because different ethnic groups may differ in response to the same stressor [31], the analyses were performed separately for each ethnic group. The associations between neighbourhood stressors and BP levels were determined using multilevel linear regression models with individuals at the first level and neighbourhoods at the second level using SAS Proc mixed procedure [32]. The associations were assessed by using beta coefficients (95 percent confidence intervals) in the fixed-effects part of the models. We also performed a multilevel logistic regression to determine the associations between neighbourhood stressors and hypertension using the SAS GLIMMIX macro procedure [33]. The results are shown as odds ratios and 95% confidence intervals. The method of estimation was a restricted maximum likelihood procedure. We performed two models to determine the associations between neighbourhood stressors and BP and hypertension adjusting for potential confounding factors. Model 1 included each neighbourhood variable and the individual level variables age and sex. In model 2 the same variables were included but in addition the individual level variables education level and BMI, which are know to be associated with BP, were added. Overweight and obesity are highly prevalent among Turkish and Moroccan ethnic groups in the Netherlands [34].

## Results

About 95 per cent of the Turkish and the Moroccan ethnic groups were first generation migrants. Table 2 shows the characteristics of the study population in each ethnic group. Turkish and Moroccan ethnic groups were younger, had lower education levels and higher BMI compared with their Dutch counterparts. Mean systolic and diastolic levels and prevalence of hypertension were lower in Turkish and Moroccans than in Dutch. These differences remained after adjustment for age and gender except for diastolic BP in Turkish people [34].

**Table 2:** Individual characteristics of the study population by ethnic group

	**Dutch**	**Turkish**	**Moroccan**
Number of participants	517	440	365
Age (yrs)	45.5 (16.8)	37.3 (13.0)	37.8 (14.6)
			
**Education %**			
Primary school or lower	7.3	42.2	30.1
Lower secondary/vocational	25.1	23.6	33.3
Intermediate vocational	27.3	24.1	27.0
Higher	40.3	10.1	9.6
			
BMI	25 (4.3)	28.1 (5.4)	27.2 (5.6)
Systolic BP	134.5 (22.3)	123.2 (18.2)	125.0 (19.6)
Diastolic BP	81.9 (11.2)	79.1 (10.8)	77.4 (10.2)
Hypertension %	41.8	24.2	23.3
			
**Neighbourhood stressors**			
% Crime			
Low	48.5 (3.6)	49.8 (3.4)	49.7 (3.5)
High	55.9 (1.4)	55.8 (1.4)	55.8 (1.4)
% Housing density			
Low	45.8(9.3)	44.8 10.7)	45.1 (10.0)
High	78.7(9.2)	74.1 (9.5)	74.0 (8.8)
% Nuisance from motor traffic			
Low	22.9 (10.6)	29.5 (9.5)	26.7 (11.1)
High	54.3 (15.0)	52.0 (13.5)	50.1 (11.8)
Mean Quality of green facilities			
Low	5.9 (0.2)	5.9 (0.2)	6.0 (0.2)
High	6.9 (0.3)	6.7 (0.3)	6.8 (0.3)
% Nuisance from alcohol misuse			
Low	5.4 (3.8)	4.7 (3.9)	4.8 (3.9)
High	29.7 (7.0)	26.3 (6.9)	25.0 (6.8)
% Nuisance from drug misuse			
Low	12.0 (4.4)	11.5 (4.0)	11.3 (3.9)
High	31.4 (7.1)	30.6 (7.4)	28.5 (7.2)
% Social participation			
Low	37.2 (5.0)	37.3 (4.5)	37.8 (4.9)
High	53.8 (7.8)	55.1 (9.2)	56.6 (8.9)

BMI = Body mass index; BP = Blood pressure

### Neighbourhood stressors and systolic blood pressure

Table 3 shows systolic BP by neighbourhood stressor in each ethnic group. Among Moroccans, high density housing and nuisance from drug misuse were associated with a higher age and sex adjusted systolic BP. In contrast, high quality of green space and high social participation were associated with a lower age and sex adjusted systolic BP. These associations persisted after further adjustment for individual-level educational level and BMI in the full model. No significant associations were noted in the Dutch and Turkish ethnic groups although the directions of the associations were similar in all ethnic groups.

**Table 3:** Multi-level regression analysis of systolic blood pressure by ethnic group and neighbourhood attributes

	Model I **Age and sex adjusted**			Model II **Adjusted for age, sex and education level and BMI**		
	**Dutch** Beta* [95% CI]	**Turkish** Beta* [95% CI]	**Moroccan** Beta* [95% CI]	**Dutch** Beta* [95% CI]	**Turkish** Beta* [95% CI]	**Moroccan** Beta* [95% CI]

**Neighbourhood attributes**						
High crime	0.49 [-3.40, 4.37]	1.50 [-2.44, 4.46]	3.32 [-1.66, 8.29]	1.03 [-2.88, 4.93]	1.11 [-2.81, 5.02]	3.31 [-1.59, 8.21]
High density housing	1.80 [-2.13, 5.73]	2.00 [-1.99 5.98]	4.83 [0.54, 9.13]*	2.75 [-1.20, 6.70]	1.58 [-2.36, 5.52]	4.79 [0.50, 9.07]*
Nuisance from motor traffic	0.63 [-3.22, 4.47]	0.96 [-2.97, 4.88]	3.09 [-1.91, 8.08]	1.27 [-2.58, 5.13]	0.46 [-3.42, 4.36]	3.01 [-.91, 7.91]
Nuisance from alcohol misuse	1.91 [-2.20, 6.10]	3.22 [-0.86, 7.30]	2.41 [-3.03, 7.85]	2.83 [-1.29, 6.96]	2.91 [-1.14, 6.95]	2.59 [-2.72, 7.91]
Nuisance from drug misuse	1.63 [-2.25, 5.51]	2.47 [-1.47, 6.42]	4.41 [0.02, 8.82]*	2.29 [-1.59, 6.17]	2.31 [-1.60, 6.22]	4.54 [0.28, 8.80]*
High quality of green spaces	-2.24 [-6.20, 1.73]	-2.13 [-6.12, 1.86]	-4.88 [-9.15, -0.60]*	-3.01 [-6.98, 0.96]	-1.72 [-5.68, 2.23]	-4.92 [-9.21, -0.64]*
High social participation	-2.14 [-6.01, 1.73]	-2.33 [-6.29, 1.63]	-5.43 [-9.82, -1.05]*	-1.72 [-5.66, 2.23]	-2.40 [-6.31, 1.52]	-5.27 [-9.67, -0.87]*

Betas represent difference in mean systolic BP; *P < 0.05, **P < 0.01 compared with lower neighbourhood attribute in each ethnic group, BMI = Body mass index.

### Neighbourhood stressors and diastolic blood pressure

Among Turkish, high crime and nuisance from motor traffic were associated with a higher diastolic BP in both models (Table 4). Among Moroccans, nuisance from drug misuse was associated with a higher diastolic BP in the full model. In contrast, neighbourhood high social participation was associated with a lower diastolic BP although the association was no longer statistically significant after further adjustment for education and BMI. Similar directions of associations were also noted in the Dutch group but none of the differences were statistically significant.

**Table 4:** Multi-level regression analysis of diastolic blood pressure by ethnic group and neighbourhood attribute

	Model I **Age and sex adjusted**			Model II **Adjusted for age, sex and education level and BMI**		
	**Dutch** Beta* [95% CI]	**Turkish** Beta* [95% CI]	**Moroccan** Beta* [95% CI]	**Dutch** Beta* [95% CI]	**Turkish** Beta* [95% CI]	**Moroccan** Beta* [95% CI]

**Neighbourhood stresses**						
High crime	0.26 [-2.42, 2.94]	3.21 [0.94, 5.47]**	0.61 [-2.63, 3.84]	0.80 [-1.74, 3.33]	2.96 [0.71 5.20]**	0.54 [-2.90, 3.98]
High density housing	-0.32 [-3.01, 2.37]	0.87 [-1.91, 3.65]	2.03 [-0.72, 4.91]	0.51 [-2.11, 3.13]	0.53 [-2.35, 3.42]	1.76 [-1.37, 4.89]
Nuisance from motor traffic	0.74 [-1.90, 3.38]	3.13 [0.87, 5.38]**	0.41 [-2.83, 3.66]	1.31 [-1.14, 3.77]	2.83 [0.60, 5.06]*	0.25 [-3.21, 3.71]
Nuisance from alcohol misuse	0.49 [-2.18, 3.16]	0.93 [-1.83, 3.69]	2.26 [-0.49, 5.00]	0.06 [-2.67, 2.79]	1.05 [-1.88, 3.98]	2.29 [-0.83, 5.42]
Nuisance from drug misuse	0.15 [-2.53, 2.83]	1.02 [-1.74, 3.78]	2.48 [-0.21, 5.17]	2.29 [-1.59, 6.17]	2.31 [-1.60, 6.22]	4.54 [0.28, 8.80]*
High quality of green spaces	1.21 [-1.37, 3.78]	-0.18 [-3.01, 2.65]	-2.17 [-4.99, 0.63]	-0.13 [-2.76, 2.52]	-0.68 [-3.57, 2.21]	-2.15 [-5.21, 0.90]
High social participation	-1.01 [-3.65, 1.63]	-0.76 [-3.53, 2.00]	-3.06 [-5.90, -0.22]*	-0.57 [-3.18, 2.04]	-0.77 [-3.61, 2.06]	-2.76 [-5.89, 0.38]

Betas represent difference in mean systolic BP; *P < 0.05, **P < 0.01 compared with low neighbourhood attribute in each ethnic group, BMI = Body mass index.

### Neighbourhood stressors and hypertension

Table 5 shows multilevel logistic regression of hypertension by neighbourhood stressor in each ethnic group. Amongst Moroccans, neighbourhoods with high quality of green space were associated with lower odds of hypertension. Similar directions of associations were also noted in the Dutch and Turkish groups but none of the differences were statistically significant.

**Table 5:** Multi-level logistic regression showing odds ratios (and 95% confidence intervals) of hypertension by ethnic group and neighbourhood stress

	Model I **Age and sex adjusted**			Model II **Adjusted for age, sex and education level and BMI**		
	**Dutch** Beta* [95% CI]	**Turkish** Beta* [95% CI]	**Moroccan** Beta* [95% CI]	**Dutch** Beta* [95% CI]	**Turkish** Beta* [95% CI]	**Moroccan** Beta* [95% CI]

**Neighbourhood stresses**						
High crime	0.94 [0.61, 1.47]	1.27 [0.81, 1.99]	1.37 [0.84, 2.23]	1.07 [0.67, 1.71]	1.18 [0.75, 1.86]	1.36 [0.82, 2.26]
High density housing	0.89 [0.58, 1.37]	1.15 [0.73, 1.79]	1.42 [0.87, 2.33]	1.10 [0.68, 1.77]	1.05 [0.67, 1.67]	1.42 [0.85, 2.37]
Nuisance from motor traffic	0.92 [0.59, 1.42]	1.23 [0.79, 1.92]	1.31 [0.81, 2.14]	1.05 [0.66, 1.67]	1.14 [0.72, 1.79]	1.30 [0.78, 2.15]
Nuisance from alcohol misuse	0.93 [0.59, 1.47]	1.39 [0.88, 2.22]	1.25 [0.75, 2.10]	1.17 [0.71, 1.92]	1.35 [0.84, 2.16]	1.29 [0.76. 2.22]
Nuisance from drug misuse	0.87 [0.57, 1.34]	1.32 [0.84, 2.04]	1.47 [0.90, 2.41]	1.02 [0.64, 1.69]	1.25 [0.79, 1.97]	1.53 [0.92, 2.55]
High quality of green spaces	1.10 [0.71, 1.74]	0.81 [0.52, 1.27]	0.62 [0.38, 1.02]	0.92 [0.57, 1.49]	0.87 [0.55, 1.38]	0.61 [0.36, 0.99]*
High social participation	0.67 [0.45, 1.00]	0.79 [0.50, 1.23]	0.62 [0.37, 1.04]	0.71 [0.46, 1.09]	0.77 [0.49, 1.22]	0.64 [0.37, 1.18]

*P < 0.05 compared with low neighbourhood attribute in each ethnic group, BMI = Body mass index.

## Discussion

Little is known about the effects of neighbourhood-level environmental stressors on BP and hypertension in different ethnic groups in Europe. Our findings show that neighbourhood-level stressors are associated with BP in ethnic minority groups but were less evident in Dutch people living in Amsterdam, the Netherlands.

Some limitations within this study should be acknowledged. As in numerous epidemiological surveys our BP levels were based on two measurements at a single visit, which might have overestimated the BP levels and the prevalence of hypertension. A further limitation was the cross-sectional nature of the study design, which indicates that causal associations can only be made with caution. In addition, our contextual stress variables were based on the overall assessment of the Amsterdam general population. It is possible that the assessment of these contextual variables might vary between the ethnic groups, which might further affect our study conclusions. Other potential sources of bias could have resulted from the relatively low response rate. Nonetheless, the response rate of the survey is comparable to several national surveys in the Netherlands [28,35], indicating that any systematic bias is unlikely. In addition, the number of people who did not receive their invitations because of incorrect residential address in the municipal registers is likely to be high due to the mobility of the population in Amsterdam. Therefore our actual response rate might be higher. Our contextual factors were based on only fifteen neighbourhoods and therefore relatively underpowered for multilevel modelling. Our contextual factors were dichotomised, which might reduce the power to detect associations. However, dichotomisation was necessary because of the structure of the data, since few neighbourhoods, and few people per neighbourhood, did not permit modelling of between neighbourhood variability in the outcomes. Nevertheless, the presence of multiple neighbourhoods did permit adequate estimation of the fixed effects of neighbourhood level variables (our main research question).

Evidence suggests that the health advantage of foreign-born people may be explained by the healthy migrant effect [36]. Nearly 95 per cent of both Turkish and Moroccans studied were first generation immigrants. It is possible that the healthy migrant effect might have underestimated the observed associations in our study.

In addition, we were unable to assess factors such as internal migration within the study area, the degree of residential segregation, and multiple dimensions of socio-economic deprivation over the life course, which might also affect our study conclusions. For example, the impact of internal migration between neighbourhoods within Amsterdam is likely to lead to underestimation of the observed associations in our study. Nevertheless, evidence suggests a weak association between selective migration and health in the Netherlands [37,38].

Despite these limitations, the study findings provide important information on the effect of environmental stressors on BP and hypertension among different ethnic groups. As far as we are aware, this is the first study that has assessed the effect of neighbourhood level stressors on BP and hypertension among ethnic minority groups. The neighbourhoods considered in our study were socio-culturally rather homogenous communities [39]. It has been emphasised that contextual or area bound factors may have a greater impact on health if a neighbourhood relates to a socio-culturally homogeneous community [30].

Our findings of associations between neighbourhood crime, nuisance from alcohol and drug misuse and BP among the ethnic minority groups add to the existing literature documenting associations between neighbourhood factors and cardiovascular risk factors [14-17,40]. For example, a recent study from Sweden showed a positive association between neighbourhood crime and CHD risk even after controlling for the individual level factors [16]. The associations between housing density, motor traffic, and BP are also consistent with recent reports [24,41]. For example, Galea and colleagues study found that living in a neighbourhood characterised by a poor quality built environment was associated with a greater likelihood of depression [24]. Also, in Glasgow, Scotland, an introduction of a traffic calming scheme resulted in improvements in health and health related behaviours in a neighbourhood with a high level of motor traffic problems [41].

Although the evidence for the associations between neighbourhood environment and cardiovascular risk factors are mounting, the explanations for these associations still remain unclear. Two main interpretations have, however, been proposed for the relative bad health of people living in disadvantaged neighbourhoods: a neo-material perspective and a psychosocial perspective. According to the proponents of the neo-material theory, impaired health of residents of some neighbourhoods is the result of accumulation of exposure and experiences that have their roots in the material world [42]. Under the proponents of psychosocial theory, stressors in the neighbourhood make residents feel unpleasant and this affects their behaviour (inappropriate coping strategies) and biology (psycho-neuro-endocrine mechanisms), which in turn, increase their susceptibility to diseases in addition to the direct effects of absolute material living standards [43].

Our findings support the psychosocial perspective and are consistent with other studies that have demonstrated associations between neighbourhood-level psychosocial factors and other health outcomes [43-45]. For example, it has been shown that a significant portion of health differentials across neighbourhoods is due to stress levels differences across neighbourhoods [45]. It is possible that the biological pathway between these neighbourhoods' environment and BP may be mediated by an abnormal neuro-endocrine secretory pattern [26] due to stress, with the effect being greater in ethnic minority groups. It may also well be that ethnic minority people living in neighbourhoods with a high level of crime or nuisances from drug and alcohol misuse might feel more vulnerable or unsafe than their European counterparts to engage in important lifestyle measures that are important for hypertension prevention (such as walking).

The associations between housing density, motor traffic, and BP seem to suggest that living in neighbourhoods characterised by a poor quality built environment is associated with psychosocial stress which, in turn, may place one at greater risk for developing high BP. The reasons for the stronger associations between neighbourhood stressors and BP in the ethnic minority groups as compared with their Dutch counterparts are unclear. However, it is possible that the ethnic minority groups in this study live in more disadvantaged and stressful parts of the same neighbourhood or have less effective coping mechanisms than their Dutch counterparts, which might have contributed to the stronger associations observed in this study. These findings may also be a reflection of concentration of other deleterious elements of the neighbourhood environment that, through various mechanisms, shape BP. These findings may also reflect residential segregation, as well as differential exposure to other factors such as racism [46-49]. Studies have shown that racism is positively associated with high BP in African Americans in the USA [46-49]. Although information on racism and health is limited in the Netherlands, this possibility cannot be ruled out and it emphasises the need to explore how racism might contribute to ethnic inequalities in health [50].

In contrast, our findings show that living in a neighbourhood with a high quality of green space and social participation was associated with a lower systolic BP and lower odds of hypertension in the Moroccan group. Similar non-significant associations were also observed amongst the Dutch and Turkish ethnic groups. It is likely that the quality of neighbourhood built such as green space provides an opportunity for more outdoor recreation and encourage healthier lifestyles. Takano et al's study also found that living in a neighbourhood with greenery filled public areas positively influenced the longevity of urban senior citizens [51]. It is widely recognised that good social relationships and affiliation have powerful effects on health, possibly through information exchange and establishment of health-related group norms [25]. In Johnell et al's study, low social participation was associated with low adherence with antihypertensive therapy [52]. Our study findings are in agreement with these previous reports.

Several neighbourhood stressors were strongly associated with BP among Turkish and Moroccan people as compared with Dutch people. This may reflect concentration of multiple stressors in disadvantaged neighbourhoods where many ethnic minority people live.

The findings of this study have important public health and clinical implications. It has been estimated that reducing the mean population BP level by even as little as 2–3 mmHg could have a major impact in reducing associated morbidity and mortality [38]. For example, a 2 mmHg reduction of systolic BP at the population level would result in an 8% overall reduction in mortality due to stroke, a 5% reduction in mortality due to CHD, and a 4% decrease in all-cause mortality. A 5 mmHg reduction would result in 14% reduction for stroke, 9% for CHD, and a 7% for all-cause mortality [53]. In this present study, the neighbourhood mean systolic BP was nearly 5 mmHg higher among Moroccan people living in neighbourhoods with high density housing and nuisance from drug misuse than their counterparts living in more advantaged neighbourhoods. The mean neighbourhood diastolic BP was also nearly 3 mmHg higher among Turkish living in high crime and motor traffic nuisance neighbourhoods than their counterparts in more advantaged neighbourhoods. Given the effect of these adverse neighbourhood stressors on BP, primary prevention measures targeting these factors may have a major impact in reducing high BP related morbidity and mortality especially among disadvantaged ethnic groups in many industrialised countries. These findings may also indicate that clinical assessment and management of BP might have to consider both individual level and neighbourhood level characteristics especially among ethnic minority patients.

## Conclusion

The findings from this study show associations between neighbourhood stressors and BP among Turkish and Moroccan ethnic groups whereas no associations could be observed among the Dutch group. The findings might indicate that the higher BP levels found in some of the ethnic minority groups (such as African descent populations and Hindustanis Surinamese) in Europe and elsewhere may be partly due to their greater susceptibility to the adverse neighbourhood environment in which many minority people live. Primary prevention measures targeting these neighbourhood attributes may have an impact in reducing high BP related morbidity and mortality especially among ethnic groups.

## Competing interests

The author(s) declare that they have no competing interests.

## Authors' contributions

All were responsible for study concept and design. JU and EL were responsible for data collection. CA, CH, WW, KS and MD were responsible for analysis and interpretation of data. CA drafted the manuscript and all were involved in critical revision of the manuscript. All authors read and approved the final manuscript.

## Pre-publication history

The pre-publication history for this paper can be accessed here:


